# Multi-drug resistant *Acinetobacter* ventilator-associated pneumonia

**DOI:** 10.4103/0970-2113.71952

**Published:** 2010

**Authors:** Vishal B. Shete, Dnyaneshwari P. Ghadage, Vrishali A. Muley, Arvind V. Bhore

**Affiliations:** *Department of Microbiology, BJ Medical College and Sassoon General Hospital, Pune, India*; 1 *SKN Medical College and General Hospital, Pune, Maharashtra, India*

**Keywords:** *Acinetobacter*, multi-drug resistance, ventilator-associated pneumonia

## Abstract

**Background:**

Ventilator-associated pneumonia (VAP) due to a multi-drug resistant (MDR) *Acinetobacter* is one of the most dreadful complications, which occurs in the critical care setting.

**Aims and objectives:**

To find out the incidence of *Acinetobacter* infection in VAP cases, to determine various risk factors responsible for acquisition of *Acinetobacter* infection and to determine the antimicrobial susceptibility pattern of *Acinetobacter*.

**Materials and Methods:**

A total of 60 endotracheal aspirate specimens from intubated patients diagnosed clinically and microscopically as VAP were studied bacteriologically. All clinical details and prior exposure to antibiotics were recorded.

**Results:**

An incidence of 11.6% of *Acinetobacter* VAP cases was recorded. Various underlying conditions like head injury, cerebral hemorrhage and chronic obstructive pulmonary disease (COPD) were found to be associated with *Acinetobacter* VAP. *Acinetobacter* strains exhibited MDR pattern.

**Conclusion:**

Strict infection control measures, judicious prescribing of antibiotics, antibiotic resistance surveillance programs and antibiotic cycling should be adopted to control infections due to these bacteria in patients admitted to intensive care units.

## INTRODUCTION

Ventilator-associated pneumonia (VAP) refers to pneumonia which occurs in people who have required mechanicalnone ventilation through an endotacheal or tracheostomy tube for at least 48 hours. VAP is a common and severe complication of critical illness that is associated with an increased length of stay in the hospital or intensive care unit and with a high mortality rate.[1]

*Acinetobacter* is a nonfermenting, gram-negative, aerobic coccobacillus found extensively in natural environment that has assumed an increasing importance in nosocomial infections in general and in VAP in particular. *Acinetobacter* species are a common cause of late-onset VAP, which occurs more than five to seven days after admission to the hospital, and such species are associated with a higher mortality rate than are other bacteria.[2] *Acinetobacter* grows in moist environments and often leads to nosocomial outbreaks of pneumonia.[3]

Imipenem therapy was, once, the “gold standard” for pneumonia due to *A. baumannii*.[4] However, this microorganism has rapidly developed the resistance to the majority of antimicrobials, including aminoglycosides, fluoroquinolones, and carbapenems.[5] Therefore, the management of VAP caused by multidrug-resistant (MDR) *A. baumannii* is an ongoing challenge. We report the prevalence of *Acinetobacter* infection in VAP cases, associated risk factors for *Acinetobacter* infection and antimicrobial susceptibility pattern of *Acinetobacter* isolated from intubated patients with pneumonia admitted to intensive care unit. (ICU)

## MATERIALS AND METHODS

The study, conducted during the years 2005 and 2006, includes patients of pneumonia who were on mechanical ventilation for more than 48 hours and admitted to the intensive care unit. To diagnose VAP in these patients, clinical pulmonary infection score[6] was employed. Patients were screened for the following parameters:

1) New or persistent pulmonary infiltrates not otherwise explained appeared on chest radiographs; 2) Fever; 3) Leucocytosis; 4)Oxygenation: PaO_2_/FiO_2_, and 5) Purulent respiratory secretions. Endotracheal aspirates from the suction trap of these patients were studied bacteriologically to find out the incidence of *Acinetobacter* VAP.

The aspirates were processed by a standard microbiological procedure.[7] The aspirate specimens showing presence of 25 polymorphonuclear (PMN) leucocytes per high power field (HPF) on Gram stain were included in the study. Such 60 microscopically suggestive VAP aspirate specimens from 60 patients were studied microbiologically.

Semi-quantitative culture method[8] was followed.Four-quadrant method was used. The purulent aspirates screened by Gram stain were washed three times in 0.9% sterile saline before inoculation on blood agar, cysteine lactose electrolyte-deficient agar. Growth was graded as no growth, +1, +2, +3, +4.

Identification of *Acinetobacter* species was made on the basis of phenotypic criteria recommended by Gerner-Smidt.[9,10] (Gram staining, colony morphology, penicillin susceptibility, oxidase, catalase and urease activity, citrate reduction, gelatin hydrolysis, glucose and lactose fermentation, and growth at 37°C and 44°C etc.) The VAP cases where PMN>25/HPF in the tracheal aspirate smears and *Acinetobacter* showing significant growth were labeled as ‘*Acinetobacter* VAP’. Various associated conditions in these patients were noted. Antibiotics given before acquisition of VAP were recorded.

Antimicrobial susceptibility testing was performed on Muller Hinton agar by disc diffusion method for the following antimicrobial agents according to the Clinical and Laboratory Standards Institutes (CLSI) guidelines:[11]

Amikacin (30 μg), ampicillin (10 μg), cefotaxime (30 μg), ceftazidime (30 μg), ciprofloxacin (5 μg), gentamicin (10 μg), tetracycline (30 μg), chloramphenicol (30 μg), co-trimoxazole (25 μg), imipenem (10 μg), meropenem (10μg).

*Escherichia coli* ATCC 25922 and *Pseudomonas aeruginosa* ATCC 27853 were used as quality control strains.

## RESULTS

Of the total aspirate specimens received, only those specimens suggestive of VAP microscopically were subjected to bacteriological studies; 60 aspirate specimens were studied. A total of 7 *Acinetobacter* species were isolated from these specimens. All the isolates were identified as *Acb complex*.

Various associated clinical conditions and history of antibiotics received before commencement of VAP in these seven patients were studied. Chronic obstructive pulmonary disease was found to be associated in four out of seven patients. The findings are displayed in Table 1

**Table 1:** Associated clinical condition and details of antibiotics received prior in *Acinetobacter* VAP patients

Associated clinical condition	No. of patients
Head trauma	2
Cerebral haemorrhage	1
COPD	4
Antibiotics received before commencement of VAP	
Cefotaxime and amikacin	4
Cefotaxime and gentamicin	2
Ceftazidime and amikacin	1

VAP: Ventilator-associated pneumonia; COPD: chronic obstructive pulmonary disease

Prior exposure to third generation cephlosporins and aminoglycisides was observed in all seven patients. Four out of seven (57.1%) patients grew *Acinetobacter* on blood culture.

The disc diffusion susceptibility result for seven *Acb complex* strains is given in Table 2. It shows the resistance percentages of *Acb complex* against the various antimicrobial agents tested. Higher resistance percentages were observed against ampicillin, third and fourth generation cephalosporins, fluoroquinolones and aminoglycosides. 100% sensitivity was observed against carbapenems.

**Table 2:** Resistance percentages of *Acb complex* against antimicrobial agents tested

Antibiotic	Resistance percentages *Acb complex* (*n*=7)
Amikacin	42.8 (3)
Ampicillin	100 (7)
Cefepime	85.7 (6)
Cefotaxime	71.4 (5)
Ceftazidime	71.4 (5)
Ciprofloxacin	57.1 (4)
Gentamicin	71.4 (5)
Imipenem	0
Meropenem	0
Tetracycline	57.1 (4)

Figures in parenthesis indicate the number of *Acb complex* strains

## DISCUSSION

The incidence of nosocomial pneumonia (NP) in ventilated patients is high, ranging from 7% to more than 40%. Such nosocomially acquired infections prolong hospital stay and contribute to ICU patient mortality.[12] *Acinetobacter* species are aerobic gram-negative coccobacilli that have emerged as important opportunistic pathogens, especially among critically ill patients.[13] *Acinetobacter* species are common cause of VAP. Since this organism survives in moist and dry conditions for a prolonged period, it often leads to nosocomial outbreaks.[3,14] Baraibar *et al*.[3] have reported 8.1% VAP cases caused by *Acinetobacter*. In the present study, 11.3% VAP cases could be attributed to *Acinetobacter*.

Various risk factors reported for the development of *Acinetobacter* VAP are ARDS, large-volume lung aspiration, head trauma and neurosurgery.[15] A case-control study by Baraibar *et al*,[3] examined patients who developed A baumanii infections and compared the infections to other etiologies of ventilator-associated pneumonia; the study could not detect risk factors common to both groups. In the present study, *Acinetobacter* VAP patients were found associated with various underlying clinical conditions like head trauma, cerebral haemorrhage and COPD [Table 1]. These findings suggest that intubated patients with any of the associated conditions are at increased risk of *Acinetobacter* pneumonia.

VAP due to a MDR microorganism is one of the most dreadful complications that can occur in the critical care setting. Several studies have suggested that the occurrence of VAP increases the risk of death in critically ill patients, especially when the episode of pneumonia is due to a multidrug-resistant pathogen.[2]

VAP due to MDR *Acinetobacter* has been reported by many workers.[3,12,15,16] It is well known that this organism develops rapid resistance to various groups of antimicrobials including aminoglycosides, fluoroquinolones, and carbapenems.[5] We recorded similar findings with our *Acinetobacter* strains. Resistance to majority of antimicrobial groups was observed except carbapenems [Table 2]. Resistance to imipenem is becoming more common and has recently been reported in 11% of *Acinetobacter* isolates.[17] Gladstone P *et al*,[18] from Vellore (India) reported 14.2% imipenem resistant *Acinetobacter* from patients with respiratory tract infections in ICU. However, all seven *Acinetobacter* strains in our study were sensitive to meropenem and imipenem [Table 2].

The use of fluroquinolones has been associated with the emergence of MDR *Acinetobacter*.[19] The exposure to third generation cephalosporins has been also implicated by numerous case-control studies.[15,20,21] Jean-Louis *et al*.[12] reported 22 episodes of early-onset VAP in patients receiving no prior antibiotics were caused by antibiotic-susceptible bacteria and 84 episodes of late-onset VAP in patients receiving prior antibiotics were mainly caused by potentially resistant bacteria. In the present study, all seven patients had exposure to third generation cephalosporins, this might have accounted for super infection with MDR *Acinetobacter*.

The reason for multi-drug resistance in this organism has been attributed to the intrinsic impermeability of its outer membrane and to its close relationship to the soil and aquatic environment which has made it possible for this organism to acquire highly effective resistance determinants in response to multiple challenges.[22]

To conclude, VAP due to a MDR *Acinetobacter* is one of the most dreadful complications that occur in the critical care setting. This poses serious problems in choosing the right antibiotic for the treatment of sick patients admitted into the ICU. Various strategies such as strict infection control measures, judicious prescribing of antibiotics, antibiotic resistance surveillance programs and antibiotic cycling are crucial to control infections due to these bacteria in patients admitted to ICU.
